# The use of a multi-disciplinary geriatric telemedicine service (TELEG) and its acceptance at a tertiary care centre in Malaysia

**DOI:** 10.1186/s12877-024-04676-0

**Published:** 2024-02-05

**Authors:** Chuo Yew Ting, Nur Hidayati Abdul Halim, Jia Nee Ling, Ing Khieng Tiong, Nor Izzah H. J. Ahmad Shauki, Yew Fong Lee, Nor Anizah Osman, Gin Wei Chai, Shin Han Ung, Melinda Ang

**Affiliations:** 1 Pharmaceutical Services Division, Sarawak State Health Department, Ministry of Health, Jalan Diplomatik, Off Jalan Bako, Kuching, 93350 Malaysia; 2 Sarawak Heart Centre, Ministry of Health, Kota Samarahan, Malaysia; 3 Institute for Health Systems Research, National Institute of Health, Ministry of Health, Shah Alam, Malaysia; 4 Sarawak General Hospital, Ministry of Health, Kuching, Malaysia; 5 School of Medical and Life Sciences, Sunway University, Selangor, Malaysia

**Keywords:** Geriatric, Telemedicine, Sustainability, Acceptance, Use

## Abstract

**Background:**

The COVID-19 pandemic has fueled the widespread adoption of telemedicine in healthcare, particularly in Sarawak, Malaysia. This study investigates the use and acceptance of Sarawak’s inaugural multidisciplinary geriatric telemedicine service, TELEG.

**Methods:**

This cross-sectional study took place at the Sarawak Heart Centre’s geriatric department from July 1, 2021, to April 30, 2022. Convenient sampling included all TELEG-enrolled patients during this period, to achieve minimum sample size of 148. TELEG’s utilization was assessed in terms of medication therapy and treatment plan optimization, as well as enhanced healthcare accessibility. Participants’ acceptance of TELEG was measured using the Service User Technology Acceptability Questionnaire (SUTAQ) administered through Google Forms. Descriptive statistics percentages illustrated the proportion of participants who found TELEG moderately to highly acceptable. Associations between baseline characteristics and overall acceptance were explored through bivariate analyses, including Pearson’s correlation test, independent t-test, and ANOVA. The influence of six SUTAQ dimensions on overall acceptance, multivariable linear regression using enter method was employed. Statistical significance was determined by p-values less than 0.5.

**Results:**

Among 180 geriatric patients enrolled in TELEG during the study period, 149 agreed to participate. TELEG led to medication therapy optimization for 88.6% of participants, primarily involving dose adjustment (44.7%), de-prescribing (31.8%), and prescribing (15.9%). Additionally, 53.8% received treatment plan optimization, predominantly in the form of self-care education (56.3%), referrals for further treatment (33.8%), additional laboratory investigations (29.6%), and increased monitoring (26.8%). Among those educated in self-care (*n* = 40), dietary intake (27.5%), lower limb exercise (25.0%), and COVID-19 vaccination (12.5%) were the most common topics. All participants expressed moderate to high acceptance of TELEG (mean = 4.9, SD = 0.65, on a scale of 1 to 6). Notably, care personnel concern (B = 0.256; *p* < 0.001) had the most significant impact on overall acceptance.

**Conclusion:**

This pioneering study evaluates the utilization and user acceptance of a geriatric telemedicine service in the region, providing valuable insights to support its expansion. Follow-up surveys or interviews to gain insights into users’ experiences are crucial to further enhance acceptance.

**Supplementary Information:**

The online version contains supplementary material available at 10.1186/s12877-024-04676-0.

## What is already known?

• Telemedicine among the elderly has also shown significant economic benefits by reducing medical costs and societal costs.

• Telemedicine among geriatric patients had been found to improve their quality of life, enhance accessibility to healthcare services, reduce hospitalization rates, and facilitate early detection of abnormalities among the patients.

**Supplementary Information:**

The online version contains supplementary material available at 10.1186/s12877-024-04676-0.

## What this paper adds?

• This is the first paper that evaluate the use and acceptability of geriatric telemedicine in a developing country in the Southeast Asia region.

• TELEG is the leading multi-disciplinary geriatric telemedicine service implemented in Malaysia.

• The findings are important for policymakers in revising the existing legal and regulatory framework to govern the implementation and expansion of geriatric telemedicine in the region.

## Background

The adoption of telemedicine has accelerated worldwide during the COVID-19 pandemic [1, 2]. Telemedicine is particularly crucial for the elderly, who often experience frailty, multiple comorbidities, and require close monitoring to ensure continuous care [3]. Studies have shown that telemedicine among geriatric patients can enhance their quality of life, improve accessibility to healthcare services, reduce hospitalization rates, and facilitate early detection of abnormalities [4]. Additionally, telemedicine among the elderly has demonstrated significant economic benefits by reducing both medical and societal costs [5–8].

Despite the rapid expansion of telemedicine during the pandemic, research on the acceptance and benefits of telemedicine in Malaysia remains limited. In Malaysia, two studies have investigated the perceived acceptance of telemedicine among healthcare providers [9, 10], while one study examined the perceived acceptance among healthcare recipients [11]. Notably, a recent pilot study found that telemedicine provided by the cardiac clinic was highly accepted by both elderly patients and caregivers [12]. Given the variations in telemedicine implementation across facilities and countries, it is crucial to assess the feasibility and acceptance of any newly implemented telemedicine systems.

Recently, there has been growing trend in adopting hybrid telemedicine as a future healthcare model, combining virtual clinics with in-person visits [13]. This hybrid approach is particularly practical in developing countries where innovative and advanced information and communication technology (ICT) infrastructure is still lacking, along with integration into existing health information systems, which are essential for telemedicine implementation.

In June 2020, the geriatric department of Sarawak Heart Centre (SHC) [14] introduced a hybrid telemedicine service for geriatric patients known as TELEG. SHC, as the sole public cardiology referral centre in Sarawak, houses the only geriatric department serving geriatric patients referred from healthcare facilities across Sarawak. The geriatric care team at SHC, led by two geriatricians, includes medical officers, pharmacists, nurses, dietitians, occupational therapists, and physiotherapists. SHC has been designated as a special institute under the Ministry of Health, Malaysia since 2015 [14]. Currently, it has 20-bed ward and caters to approximately 1500 geriatric patients in Sarawak.

The initial implementation of TELEG aimed to benefit geriatric patients at a high risk of COVID-19 infection, those facing long travel distances to SHC, and those unable to travel due to movement control orders. Notwithstanding, the overarching goal of TELEG is to enhance healthcare services accessibility and ensure the continuity of geriatric care. As TELEG is a recent addition, its utilization within the existing treatment model and its acceptance among users remain unknown.

The objective of this study is to investigate the use of telemedicine in optimizing medication therapy and care plans, improving healthcare service accessibility, and assessing patient acceptance of TELEG. The study findings will provide valuable insights for policymakers, relevant authorities, and healthcare professionals, on how to implement telemedicine that would be beneficial and well-accepted.

## Methods

A cross-sectional study examining the use of TELEG and patients’ acceptance was conducted from June 2021 to May 2022 at the geriatric clinic of SHC.

### Sample size and sampling method

Data from the geriatric department at SHC showed that approximately 20 geriatric patients were enrolled in TELEG every month. It was assumed that 50% of the participants would find TELEG moderately to highly acceptable. This 50/50 assumption was made due to limited or nonexistent studies reporting the acceptance of geriatric care delivered through telemedicine among the elderly population in Malaysia. As this is a finite population, the formula used for sample size estimation was: n = [NZ^2^ * p(1 − p)] / [d^2^ * (N − 1) + Z^2^p(1-p)]. By determining Z = 1.96 for a 95% confidence interval (CI) and c = 0.05 margin of error, *p* = 0.5, the minimum sample size was calculated to be 148 [15]. Considering a 20% of non-response rate, a minimum of 178 participants were targeted. Convenient sampling was employed to invite all patients enrolled in TELEG during the study period to participate.

The inclusion criteria for TELEG participants were as follows: (1) They had been seen at least once physically by the attending physician (either at the wards or geriatric clinic); (2) Either the patients or their caregivers had a smartphone that supported video conferencing; (3) They had a stable internet connection at their homes; (4) They had impaired mobility or were bed-bound; (5) They were clients of a nursing home; (6) They required monitoring of side effects after initiating drugs.

The exclusion criteria for TELEG participants were as follows: (1) Declining to provide informed written consent; (2) Requiring physical examinations; (3) Being new cases to the geriatric clinic.

### Ethical approval and participants recruitment process

This study received approval from the Medical Research and Ethics Committee (MREC), National Institutes of Health Malaysia, before its commencement. Informed consent was obtained from patients and/or their caregivers upon their agreement to participate in TELEG. All patients enrolled in TELEG during the data collection period were approached, and informed written consent was administered through Google Form (Supplementary material 1). This ensured that patients were informed about the study’s objectives, procedures and their right to withdraw before participating. Furthermore, the procedures for using their smartphones to access TELEG were explained and demonstrated.

**Fig. 1:**
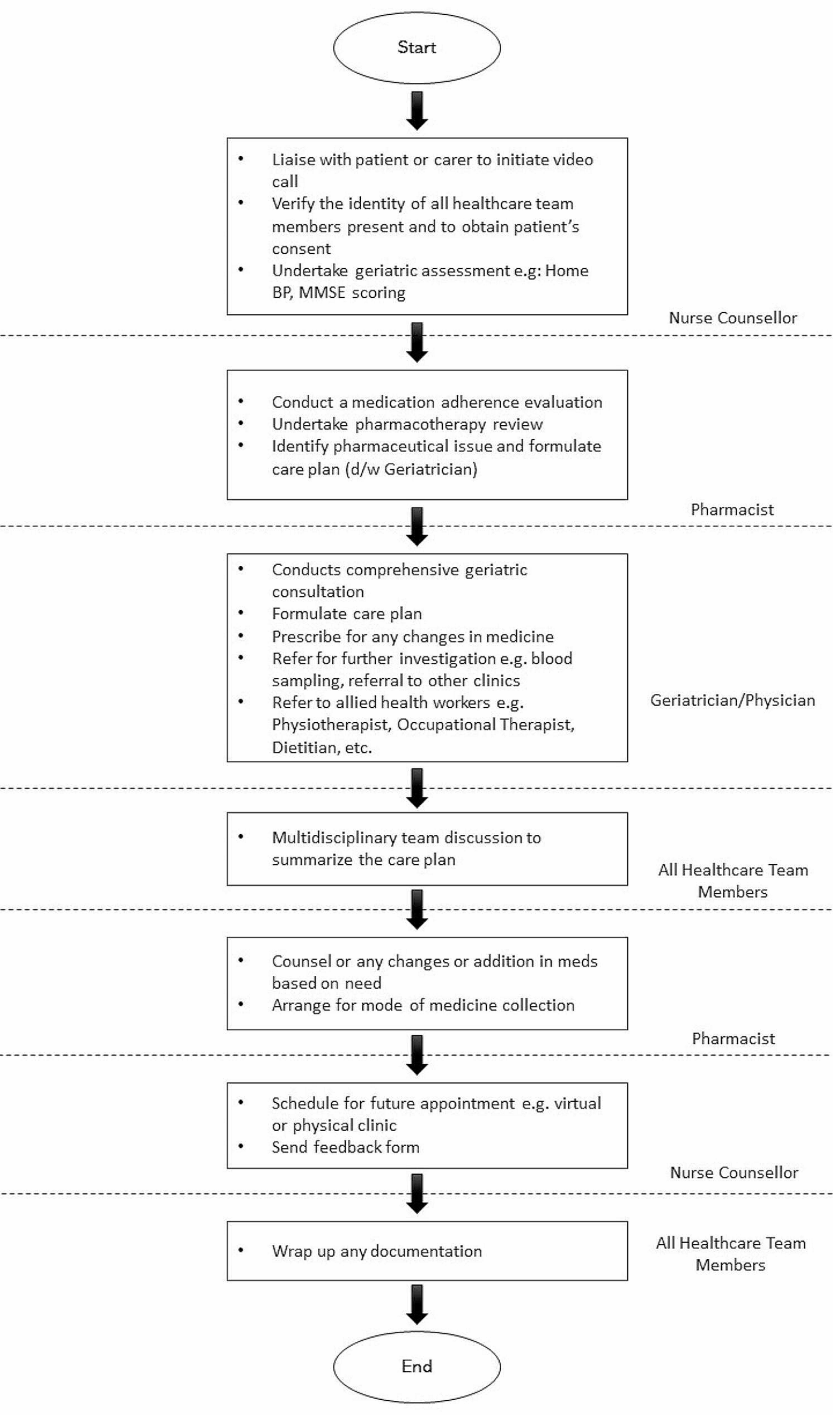
Flowchart of TELEG care model

One week prior to a TELEG consultation, patients and/or their caregivers received a reminder and the appointment link. On the day of the appointment, a reminder was sent again by the nurse in charge of TELEG. During the consultation, patients were registered via the hospital system, and the nurse initiated the connection via Zoom or Google Meet, awaiting the patient’s presence. The flow of TELEG is illustrated in Fig. 1. At the end of the session, patients had the option of medication dispensing via post or self-collection at the counter. The doctor decided the patient’s next appointment, either to continue with TELEG or scheduled a physical clinic visit, with the appointment date provided by the nurse. The entire session typically lasted less than an hour.

### Study outcomes and instrument

In this study, the optimization of medication therapy was quantified based on the count of medication prescriptions, de-prescriptions, or dosage adjustments for each patient during their first TELEG session [16, 17]. For example, if a prescriber both prescribed a new medication and adjusted the dose for a patient, it would contribute to one count each of a prescribed new medication and a dosage adjustment in the optimization of medication therapy.

Similarly, the optimization of the care plan was quantified based on the count of action plans for each patient, including recommendations for further laboratory examinations, referrals to other healthcare professionals, and patients’ self-care education. For instance, if a geriatrician advised a patient to undergo further laboratory examinations and provided self-care education, it would contribute to one count each of further laboratory examinations and patient self-care education. This information was recorded using Google Forms (Supplementary material 2).

Physical accessibility was calculated by comparing the two-way driving distance and time for patients to travel to SHC versus attending TELEG [18]. Google Earth Pro was used to measure the driving distance and time from the residential address to SHC. A positive difference indicated an improvement in physical accessibility through TELEG, while a negative difference suggested the opposite.

Acceptance of TELEG was assessed in terms of its benefits, perceived privacy, personal care skill of the healthcare providers, TELEG as substitution and user satisfaction using the Service User Technology Acceptability Questionnaire (SUTAQ) [19]. SUTAQ was chosen as it was specifically designed to evaluate the acceptance of telemedicine services [20]. Its validity among elderly patients with chronic diseases made it suitable for this study. Additionally, patients were interviewed to gain further insights into their acceptance to TELEG.

SUTAQ comprises six dimensions with a total of 22 items: (1) enhanced care (5 items), (2) increased accessibility (4 items), (3) privacy (4 items), (4) care personnel concerns (3 items), (5) substitution (3 items), and (6) satisfaction (3 items). Respondents rated their agreement using a 6-point Likert scale, with 6 indicating “strongly agree” and 1 indicating “strongly disagree”. The reliabilities of the six dimensions of the original SUTAQ on enhanced care, increased accessibility, privacy, care personnel concern, substitution, and satisfaction are 0.831, 0.830, 0.707, 0.630, 0.642, and 0.766, respectively. It was posited that the perceived benefits, increased accessibility, privacy and comfort, care personnel concerns, satisfaction, and telemedicine as substitution, have significant influences on the overall acceptance towards TELEG.

SUTAQ, originally in English (Supplementary file 3), was translated into Bahasa Malaysia (National Malaysian Malay language) and Mandarin to accommodate respondents who were not fluent in English. It was the back-translated into English by two experienced language lecturers from the Faculty of Language and Communication, University of Malaysia Sarawak. The content of the translated SUTAQ was independently evaluated by two public health experts from the University of Malaysia Sarawak. Any discrepancies between the two experts were resolved through discussion led by the principal investigator.

Using Google forms, data were collected during the first TELEG session. The cross-sectional study included eight socio-demographic characteristics: age, gender, highest education level, residential area, living status, place of stay, source of financial expenses, and the main caregiver. Patients’ health status was assessed based on comorbidities, the level of frailty using the Clinical Frailty Scale (CFS), and pressure ulcer staging (if present upon enrolment). The CFS used in this study [21, 22] has been shown to accurately predict frailty, comorbidity, and disability for mortality over a 1-year follow-up in hospitalized geriatric patients. The most recent version of the CFS published by Rockwood & Theou [22] was adopted in the study. It was a 9-point single item measure with 1 indicated “Very fit”; 2 indicated “fit”; 3 indicated “Managing well”; 4 indicated “Living with very mild frailty”; 5 indicated “Living with mild frailty”; 6 indicated “Living with moderate frailty”; 7 indicated “Living with severe frailty”; 8 indicated “Living with very severe frailty”; 9 indicated “Terminally ill”. Permission to use the CFS was obtained from Prof. Dr. Rockwood of Dalhousie University, Canada. SUTAQ was also administered using Google Forms. An overview of the data collection form is presented in Table 1.

**Table 1:** Data collection form used in the cross-sectional study

Variables	Number of items	Measure	References
**Section A** (Administered by researcher/nurse)			
Socio-demographic characteristics	9	(1) Age (1 item) (2) Gender (1 item) (3) Highest education level (1 item) (4) Residential area (2 items) (5) Living status (1 item) (6) Place of stay (1 item) (7) Source of financial expenses (1 item) (8) Main caregiver (1 item)	Disclosed by the patients upon enrolment to the study
**Section B** (Administered by researcher/nurse)			
User’s acceptance	22	Service User Technology Acceptability Questionnaire (SUTAQ)	Hirani et al., 2017
**Section C** (Administered by geriatrician)			
Comorbidities	1	As written in the medical card	Medical card
Level of frailty	1	9-point single item measure. 1 = Very fit; 2 = fit; 3 = Managing well; 4 = Living with very mild frailty; 5 = Living with mild frailty; 6 = Living with moderate frailty; 7 = Living with severe frailty; 8 = Living with very severe frailty; 9 = Terminally ill.	Rockwood et al. 2005; Rockwood & Theou, 2020
Pressure Ulcer Staging (If applicable)	1	Revised Pressure Injury Staging System (RPISS). The RPISS has six stages, including 1, 2, 3, 4, non-stageable and deep tissue injury. The lower stage indicates less severe pressure ulcer	Edsberg et al., 2016
**Section D** (Administered by pharmacist/nurse)			
Appropriate use of medication	1	Assessing the knowledge of patient/caregiver on the dose, frequency, indication, and route of administration for all the medications taken by the patients.	Wei et al., 2020

### Data collection confidentiality

As aforementioned, data were collected using Google Form. Once retrieved from Google Forms, the data were recorded in offline Microsoft Excel spreadsheets. Subsequently, all data within the Google Form were permanently deleted, using the “delete forever” option within the “Trash” folder of administrator’s Google Drive. This deletion was carried out to ensure the confidentiality of patient data. The data obtained from this study will not enable the identification of individual patients, and only aggregated data will be analyzed and published.

### Data analysis

The data collected through Google Forms were exported into Microsoft Excel. To ensure data quality, duplicates and invalid responses were filtered out, and missing data were handled using the principle of listwise analysis. To determine the proportion of participants who considered TELEG as moderately to highly acceptable, descriptive statistics, specifically percentage, were used to present the findings. The associations between numerical baseline characteristics and overall acceptance (overall SUTAQ score) were examined using Pearson’s correlation test. For categorical baseline characteristics, associations with overall acceptance were assessed using either independent t-tests or ANOVA tests, depending on the number of groups within the independent variable.

As mentioned previously, SUTAQ comprises six dimensions. The average overall SUTAQ score and the average scores for each SUTAQ dimension were calculated. To explore the influence of six dimensions of SUTAQ on overall acceptance, multiple linear regression analysis using the enter method was employed. Multicollinearity was assessed (variance inflation factor < 5), and the normality of residuals was checked using histograms and normal P-P plots of regression standardized residuals. All inferential statistics with *p-values* less than 0.5 were considered statistically significant. All data analyses were carried out by Statistic Package for the Social Science (SPSS) version 26.

## Results

### Patient characteristics

**Fig. 2:**
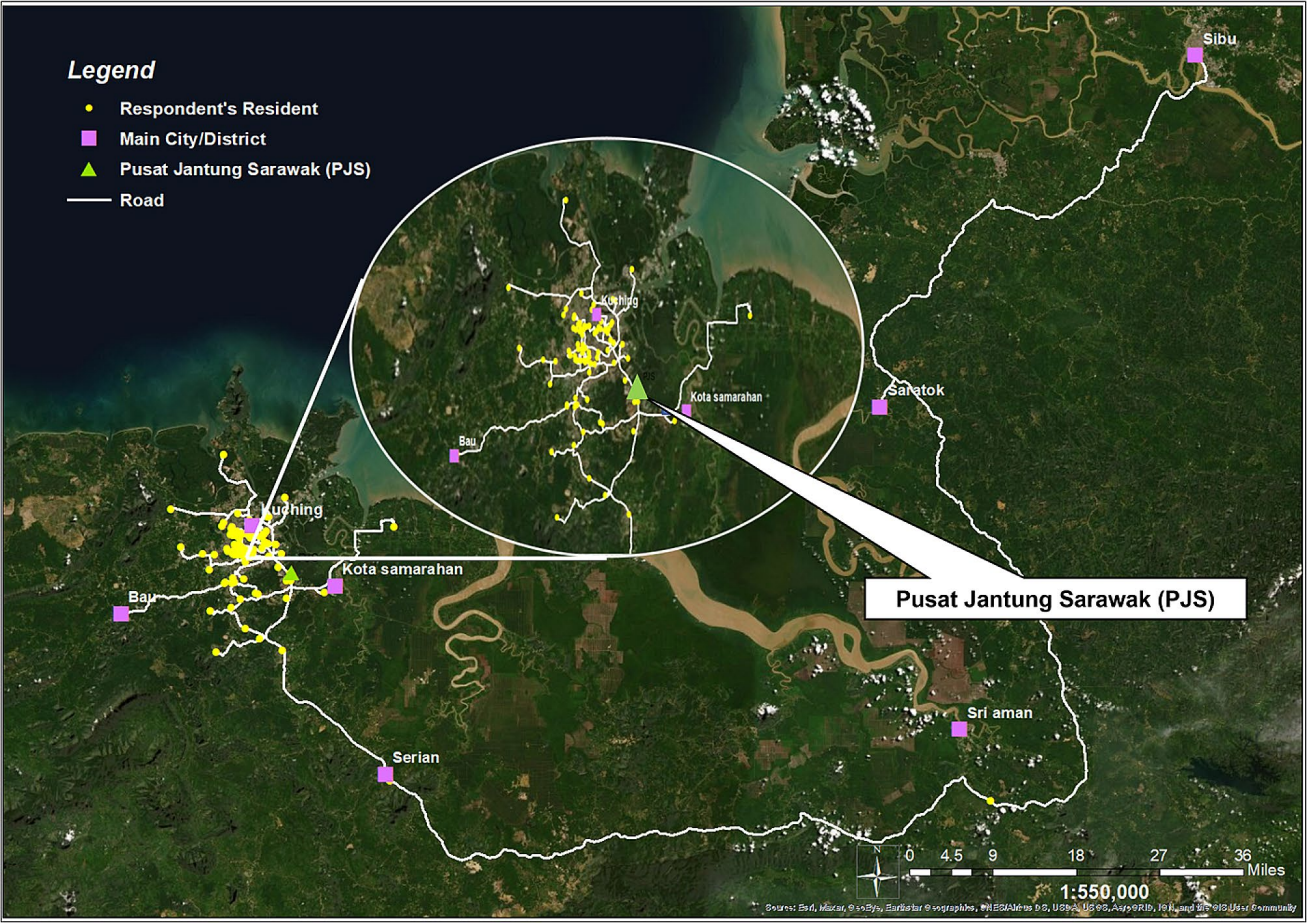
Geological mapping of respondents’ residence (in yellow dots) and Sarawak Heart Centre (in green triangle)

A total of 180 geriatric patients enrolled in TELEG during the study period, with 149 consenting to participate in the study (see Table 2). The mean age of respondents was 78.8 (± 7.71), most were female (59.7%), had not received tertiary education (96%), resided with a spouse and other family members (79.9%), and had children as their primary caregivers (43.6%). A significant portion of respondents had a clinical frailty scale score of 5 or higher (91.3%) (Table 2). The geographical mapping of respondents’ residences and the route to Sarawak Heart Centre is depicted in Fig. 2.

**Table 2:** Socio-demographic and health-related characteristics of the respondents and their associations with the overall acceptance of TELEG (SUTAQ score; *N* = 149)

Respondents’ characteristics		N (%)	Association with SUTAQ score^*^ r/t/F value^**^; P value
Socio-demographic characteristics			
Age in years	Mean (SD) = 78.8 (7.71)		−0.004; 0.958^a^
Gender	Male Female	60 (40.3) 89 (59.7)	0.957; 0.340^b^
Highest education level	No formal education Primary education Secondary education Tertiary education	49 (32.9) 51 (34.2) 43 (28.9) 6 (4.0)	0.378; 0.770^c^
Division of the residential address	Kuching Samarahan	138 (92.6) 4 (2.7)	n/a
	Serian	3 (2.0)	
	Sri Aman	1 (0.7)	
	Betong	1 (0.7)	
	Sibu	1 (0.7)	
	Miri	1 (0.7)	
Two-ways driving distance between home and SHC (KM)	Median (IQR) = 26.8 (13.6)		0.080; 0.331^a^
Average time spent on TELEG	Mean (SD) = 36.4 (17.07)		
Average time spent for physical clinic visit	Mean (SD) = 163.4 (72.39)		
Living status	Alone	4 (2.7)	0.136;
	With spouse only	5 (3.4)	0.936^c^
	With family members	119 (79.9)	
	At nursing home	21 (14.1)	
Main source of finance	Own saving/pension money	59 (39.6)	0.358;
	Money from children	81 (54.4)	0.835^c^
	Money from third parties	9 (6.0)	
Main caregiver	Spouse	43 (28.9)	0.304;
	Children	65 (43.6)	0.870^c^
	Formal caregiver	33 (22.1)	
	Sibling or relatives	4 (2.7)	
	No caregiver	4 (2.7)	
**Health related characteristics**			
Clinical Frailty Scale Scoring [22]	2 3	2 (1.3) 2 (1.3)	2.60; 0.116^c^
	4	9 (6.0)	
	5	44 (29.5)	
	6	57 (38.3)	
	7	27 (18.1)	
	8	8 (5.4)	
Top five comorbidities	Hypertension	98 (65.8)	n/a
	Hyperlipidemia	61 (40.9)	
	Diabetes mellitus	58 (38.9)	
	Dementia	58 (38.9)	
	Parkinson	17 (11.4)	
Having bedsore	Yes No	19 (12.8) 130 (87.2)	n/a

KM: Kilometers; n/a: not applicable; SD: Standard deviation; SHC: Sarawak Heart Centre; SUTAQ: Service User Technology Acceptability Questionnaire

*The associations between numerical baseline characteristics with overall acceptance (SUTAQ score) were examined using Pearson’s correlation test. For the associations between categorical baseline characteristics with overall acceptance were examined using independent t-test or ANOVA test (for independent variable with more than 2 groups)

**r value: Pearson’s correlation coefficient r; t value: statistics value for independent t test; F value: statistics value for ANOVA test

^a^Pearson’s correlation test

^b^Independent t test

^c^ANOVA test

During TELEG sessions, a substantial proportion of participants, 88.6% (*n* = 132), received medication therapy optimization, with the majority undergoing dose adjustment (*n* = 59, 44.7%), de-prescribing (*n* = 42, 31.8%) and prescribing (*n* = 21, 15.9%) (Table 3). Additionally, 53.8% (*n* = 71) of participants received treatment plan optimization, primarily focusing on self-care education (*n* = 40, 56.3%), followed by referrals for further treatment (*n* = 24, 33.8%), additional laboratory investigations (*n* = 21, 29.6%), and more frequent monitoring (*n* = 19, 26.8%). Among those who received self-care education (*n* = 40), the most common topics included dietary intake (*n* = 11, 27.5%), lower limb exercise (*n* = 10, 25.0%), and COVID-19 vaccination (*n* = 5, 12.5%).

**Table 3:** The use of TELEG in optimization of medication therapy, treatment plan and accessibility to healthcare services (*N* = 149)

Variables	Types of intervention	Frequency (%)
Optimization of medication therapy (*n* = 132)	Prescribing	21 (15.9%)
	Dose adjustment	59 (44.7%)
	De-scribing	42 (31.8%)
Optimization of treatment plan (*n* = 71)	Self-care educations (Dietary intake; home exercise; COVID-19 vaccination)	40 (56.3%)
	Referral for further treatment by physiotherapist/occupational therapist/dietitians.	24 (33.8%)
	Further laboratory investigation	21 (29.6%)
Physical accessibility to healthcare services	Average driving distance saved (thru-fro)	46.6 km ($\pm$112.52)
	Average time saved	127.0 min ($\pm$68.38)

Overall, all participants had moderate to high acceptance of TELEG (mean = 4.9, SD = 0.65; score range = 1 to 6) (Table 4). The dimension with the highest acceptance was increased accessibility (mean = 5.35, SD = 0.64), followed by satisfaction (mean = 5.30, SD = 0.83), privacy and comfort (mean = 4.92, SD = 0.88), enhanced care (mean = 4.77, SD = 0.85), concern with care personnel (mean = 4.31, SD = 1.06), and TELEG as substitution (mean = 4.17, SD = 0.99) (Table 4).

**Table 4:** Descriptive statistics and Linear regression for six dimensions of SUTAQ with overall acceptance of TELEG (*N* = 149)

Outcome	Independent variables^a^		Multiple linear regression^a^			
		Mean (SD)	Std. coeff.	95% CI	P value	
Six dimensions of SUTAQ	Increased accessibility	5.35 (0.64)	0.227	0.204, 0.250	< 0.001	
	Privacy and comfort	4.92 (0.88)	0.251	0.233, 0.268	< 0.001	
	Concern with care personnel	4.31 (1.06)	0.256	0.239, 0.273	< 0.001	
	Satisfaction	5.30 (0.83)	0.243	0.220, 0.266	< 0.001	
	TELEG as substitution	4.17 (0.99)	0.114	0.095, 0.132	< 0.001	
	Enhanced care	4.77 (0.85)	0.239	0.214, 0.263	< 0.001	

SUTAQ: Service User Technology Acceptability Questionnaire; Std. Coeff.: Standardized coefficient

^a^Assumptions testing conducted: Multicollinearity was checked and not found (variance inflation factor < 5); Homoscedasticity normality of residuals was checked by using histogram and normal P-P plot of regression standardized residual and was found fulfilled; Model fit was checked by R square, which equals to 0.994 (standard error = 0.04); intercept (95%CI) equals to −0.127

Among these dimensions of acceptance, care personnel concern had the greatest influence on overall acceptance (B = 0.256; *p* < 0.001), followed by privacy and discomfort (B = 0.251; *p* < 0.001), users’ satisfaction (B = 0.243; *p* < 0.001), enhanced care (B = 0.239; *p* < 0.001), increased accessibility (B = 0.227; *p* < 0.001), and last with TELEG as substitution (B = 0.114; *p* < 0.001) (see Table 4). None of the background characteristics were found to have significant association with participants’ overall acceptance levels (Table 2).

## Discussion

### The use of TELEG

This study sheds light on the initial implementation of telemedicine for geriatric patients by geriatricians in Sarawak, Malaysia. As aforementioned, SHC is the sole geriatric department serving geriatric patients referred from healthcare facilities across Sarawak. It’s worth noting that only two hospitals in Malaysia had initiated telemedicine for geriatric patients in June 2020 [23]. Therefore, this study contributes valuable insights into the feasibility and acceptance of telemedicine among the elderly in Malaysia and the Southeast Asia region [12, 24, 25].

This study shows that this service delivery model has led to the optimization of medication therapy, treatment plans, and increased accessibility to healthcare services. Notably, 88.6% of TELEG participants (132 out of 149; Table 3) had their medication therapy optimized through de-scribing, prescribing, and dose adjustments.

Optimizing medication therapy is a crucial element of geriatric care, especially for patients prone to multimorbidity and polypharmacy [26–28]. In this context, the involvement of pharmacists is particularly impactful [29, 30]. The TELEG model at SHC includes a multidisciplinary geriatric care team with geriatricians, medical officers, nurses, and pharmacists. This remote consultation enhances the involvement of pharmacists early in the care process, improving accessibility and enabling timely medication reviews, thereby contributing to medication optimization. Interestingly, a rapid review of virtual geriatric clinic models found none that involved pharmacists in their teams [27]. Therefore, our study offers valuable insights into the expansion of virtual geriatric care models in the literature.

Under-prescription is also common among older individuals [28, 31], and addressing it is essential to prevent adverse outcomes, such as higher risk of cardiovascular events, worsening disability, hospitalization, and death [28]. Scholars suggested that the implementation of explicit under-prescription criteria, comprehensive assessment by geriatricians, and the involvement of clinical pharmacist are crucial to address under-prescription [28]. Furthermore, the adoption of screening tools for under-prescription, such as START, is recommended [29]. Additionally, older people have different pharmacokinetic and pharmacodynamic profiles for medications [30]. Thus, it is imperative for the geriatric care team to be alert to such differences when trying to optimize their pharmacological therapy through dose-adjustment to ensure adverse drug reactions are minimized [32, 33].

It was found that 47.7% (71 out of 149) of participants had their treatment plans optimized through self-care education, referrals for further treatment, and further laboratory investigations. Patients from rural areas, those who are frail, and those experiencing financial burdens inevitably found TELEG to be a more accessible and affordable platform for receiving health advice and education compared to traditional physical clinics [3]. Participants in this study also received timelier referrals for further consultation with physiotherapist, occupational therapists, dietitians, and additional laboratory investigations, without having to wait until their next physical visit’s appointment. Studies have found that the timely introduction of interventions through virtual geriatric clinics has significantly reduce hospitalization rates [34, 35] and reduced emergency department visits [36].

In terms of improving accessibility to healthcare services, all studies related to virtual clinic consultation highlight the advantages of time and cost savings when compared to traditional physical clinic consultations [37–39]. In fact, increasing the accessibility of healthcare services has been the core value of implementing telemedicine from the beginning [40]. The time and travel cost savings could be lifesaving for many patients, especially those who reside in suburban or rural areas, require caregivers to bring them to the clinic, have limited financial capacity, or need to spend a significant amount on travel [41–43]. For example, in the case of Sarawak, at the time of conducting this study, there were only three geriatricians in Sarawak, and all three were posted in the southern zone of Sarawak. Consequently, it was extremely costly for patients from the central and northern zones to travel to the geriatric clinic at Sarawak Heart Centre, involving both transportation and accommodation expenses [41, 42]. Similarly, the three geriatricians faced considerable costs and time constraints when traveling to hospitals throughout Sarawak to see patients, given the limited time available after accounting for travel time. For patients who required caregivers to accompany them, these costs were at least doubled. Hence, the implementation of TELEG has significantly increased the accessibility and affordability of geriatric care for the elderly population in Sarawak.

### Acceptance of TELEG

This study found that patients had moderate to high levels of acceptance of TELEG. This finding is consistent with a study conducted among geriatric patients in Kuala Lumpur, Malaysia [12]. Notably, as TELEG selected participants with impaired mobility or who were bedbound, this might result in higher acceptance of TELEG compared to those who were less frail. Notwithstanding, both studies were conducted during the COVID-19 pandemic, and patients considered TELEG a useful means to increase access to the healthcare services during the movement control order while minimizing the risk of infection.

Through virtual consultation, multidisciplinary healthcare services can be delivered more efficiently, as patients can consult with different healthcare professionals during a single virtual session. Traditionally, patients may have needed separate appointments with different healthcare professionals for in-person consultations. This posed significant challenges for frail patients who required caregiver assistance and faced difficulties covering travel costs.

While previous studies have primarily focused on the acceptance of telemedicine conducted by single health professional, particularly physicians [10, 12], more research is needed to examine the acceptance and utilization of telemedicine when conducted by multidisciplinary healthcare teams. Notwithstanding, there are limitations to TELEG as mentioned by the patients and caregivers who refused to join TELEG. Specifically, poor internet connectivity and coverage, as well as disturbance at home, may hinder the acceptance of TELEG. Users of telemedicine in developing countries or underdeveloped regions with inadequate internet infrastructure may find telemedicine inaccessible, despite its potential benefits. Additionally, some caregivers who are the children of the patients may face interruptions during telemedicine session at home because they have their own children to be taken care of at the same time.

Another interesting finding was that the dimension of acceptance that had the greatest influence on overall acceptance was care personnel concern. The item of care personnel concern with the lowest mean value was the statement “I am concerned about the level of expertise of the individuals who monitor my status via the TELEG.” One possible explanation for this, as suggested by Yang and colleagues [44], could be that some patients may find it difficult to build trust with healthcare professionals during virtual consultation. Scholars have also suggested that healthcare teams should establish an evaluation mechanism to identify patients with difficulty in establishing trust through virtual consultation [44, 45]. It is also suggested that patients who are found to have difficulty establishing trust with healthcare professionals during virtual consultation may no longer benefit from virtual consultation and should thus discontinue them.

Additionally, the privacy and discomfort were also found to significantly influence the acceptance of TELEG. A review on the ethical and legal challenges of telemedicine in the era of the COVID-19 pandemic highlighted that the majority of past studies found informed consent and autonomy to be major ethical concerns (87%). Moreover, more than half of the studies found patients consulted through telemedicine were concerned about their privacy (78%), confidentiality (57%), data protection and security (74%), and professional malpractice (70%). In TELEG, all participants were provided with information about the benefits and risks and their autonomy to withdraw from TELEG at any time through a participant information sheet approved by the ethics committee. In this study, the principal investigator permanently delete the Google Form data once it was downloaded into an offline Microsoft Excel file. This was done to ensure that patient data would not remain in a third-party cloud database. Nevertheless, it is imperative for healthcare professional to choose telemedicine digital platforms that comply with high cybersecurity standards, such as the Health Insurance Portability and Accountability Act of 1996 (HIPAA 1996) enacted in the United States of America [46]. Zoom was chosen for use in TELEG during the study period because it is the video conferencing platform that had been subscribed to by the institute during COVID-19 pandemic. Notably, the US government had permitted several video chats platforms, such as Apple FaceTime, Facebook Messenger video chat, Google Hangouts video, Zoom, or Skype, without the risk of prosecution for noncompliance with the HIPAA rules [46]. Hence, it is imperative for healthcare professionals who intend to start telemedicine to be aware of the latest relevant regulations related to the practice of telemedicine.

### Implications of study findings

The findings of this study suggest potential benefits in the use of telemedicine for optimizing pharmacotherapy and patient cares, along with its acceptance among patients. In addition to the evidences of cost benefits [5, 6] and the high acceptance observed among frontline physicians [47], telemedicine appears to hold promise for the elderly population. Notwithstanding, those adopting telemedicine should also be mindful of the associated barriers. For instance, one study reported that patients had concerns about the accuracy of diagnoses made through phone calls and found certain virtual platforms to be less user-friendly [48]. Therefore, the healthcare professions who adopt telemedicine in care delivery should receive appropriate training to address such barriers. There are some useful guidelines provided by scholars [49] and practitioners (such as the Telehealth Implementation Playbook provided by American Medical Association [50]), which should be followed by telemedicine practitioners to ensure the quality of care delivered through telemedicine. Besides, it would be beneficial to incorporate telemedicine into the curricula of healthcare professionals’ training programs. To overcome barriers related to the virtual platforms used for telemedicine, infrastructure readiness, such as better internet coverage and speed, is crucial for enhancing adoption and satisfaction with telemedicine.

Another notable barrier to the implementation and adoption of telemedicine is the ethical and legal issues surrounding it. To date, there have been no clear guidelines or regulations in Malaysia, akin to HIPAA 1996, that ensure the security of patient health information and uphold the quality standards of telemedicine. The security challenges persist when using affordable video conferencing platforms such as Zoom or Google Meet for telemedicine purposes. Additionally, as there has been no dedicated health information system developed for telemedicine use at the time of this study, commercially available cloud database like Google Drive were employed. However, the security of Google Drive can be a concern. These gaps have deterred healthcare professionals from embracing telemedicine in their services due to potential legal issues that may arise. Hence, policymakers from different ministries, such as Ministry of Communications and Digital and the Ministry of Health in Malaysia, should collaborate in establishing a legal framework for the practice of telemedicine in the nation.

### Strengths and limitations of study

The are some limitations to this study. First, most of the respondents were conveniently sampled from communities in the Kuching and Samarahan divisions, which are in close proximity to the SHC. This sampling approach was necessitated by the fact that only patients who could physically visit the SHC during the country’s movement control order, imposed during the pandemic, were invited to participate in the study. Only after the movement control order was lifted did we manage to include some participants from other divisions. Consequently, the findings of this study may not be fully generalizable to the entire geriatric population throughout Sarawak. Furthermore, TELEG selected participants with impaired mobility or those who were bedbound, which is likely to result in a higher acceptance of TELEG among this demographic. However, it’s important to note that such selection criteria were implemented to prioritize patients who require TELEG care the most during the initial phase of its implementation. Moreover, the perspective of other allied healthcare professionals, aside from pharmacists and nurses, were not captured in this study. Therefore, future research endeavors should delve further into the acceptance of virtual consultation among members of multidisciplinary healthcare teams. Lastly, this study did not furnish evidence regarding the impact of TELEG on the clinical outcomes of the participants. This is primarily because monitoring clinical outcomes necessitates patients’ physical attendance at the clinic, which was challenging during the pandemic.

## Conclusion

The utilization of TELEG has demonstrated a positive impact on optimizing pharmacotherapy, refining treatment plans, and enhancing the accessibility of geriatric care services. While further studies involving a larger population in Malaysia are required to draw conclusive findings regarding the overall acceptance of virtual consultations, existing evidence suggests that elderly individuals residing in urban areas of Malaysia find the virtual consultation services provided in the country acceptable. Strengthening the existing legal and regulatory frameworks is imperative to ensure the sustainability and expansion of telemedicine within the nation. Future research efforts may explore the feasibility and potential benefits of telemedicine in promoting healthy ageing among the elderly.

### Electronic supplementary material

Below is the link to the electronic supplementary material.

**Supplementary Material 1:** Informed consent to participate TELEG

**Supplementary Material 2:** Data collection form on health interventions prescribed through TELEG

**Supplementary Material 3:** SUTAQ in English

## Data Availability

All data generated/analysed are provided as related files.

## Abbreviations

COVID-19: Coronavirus Disease 2019
TELEG: Geriatric Telemedicine
PJS: Pusat Jantung Sarawak (Malay version of Sarawak Heart Centre)
SD: Standard deviation
SHC: Sarawak Heart Centre
USA: United States of American
