# Supplementary material for: The use of a multi-disciplinary geriatric telemedicine service (TELEG) and its acceptance at a tertiary care centre in Malaysia
Source: BMC Geriatr. 2024 Feb 5;24:133. doi: 10.1186/s12877-024-04676-0 (PMC10845621; doi:10.1186/s12877-024-04676-0)
Supplement: Supplementary file 1 — Supplementary Material 1: Informed consent to participate TELEG [file 12877_2024_4676_MOESM1_ESM.pdf]

Effect, Acceptance, and Sustainability of a Hybrid Telemedicine Service among Geriatric Patients in Sarawak Heart Centre

* Required

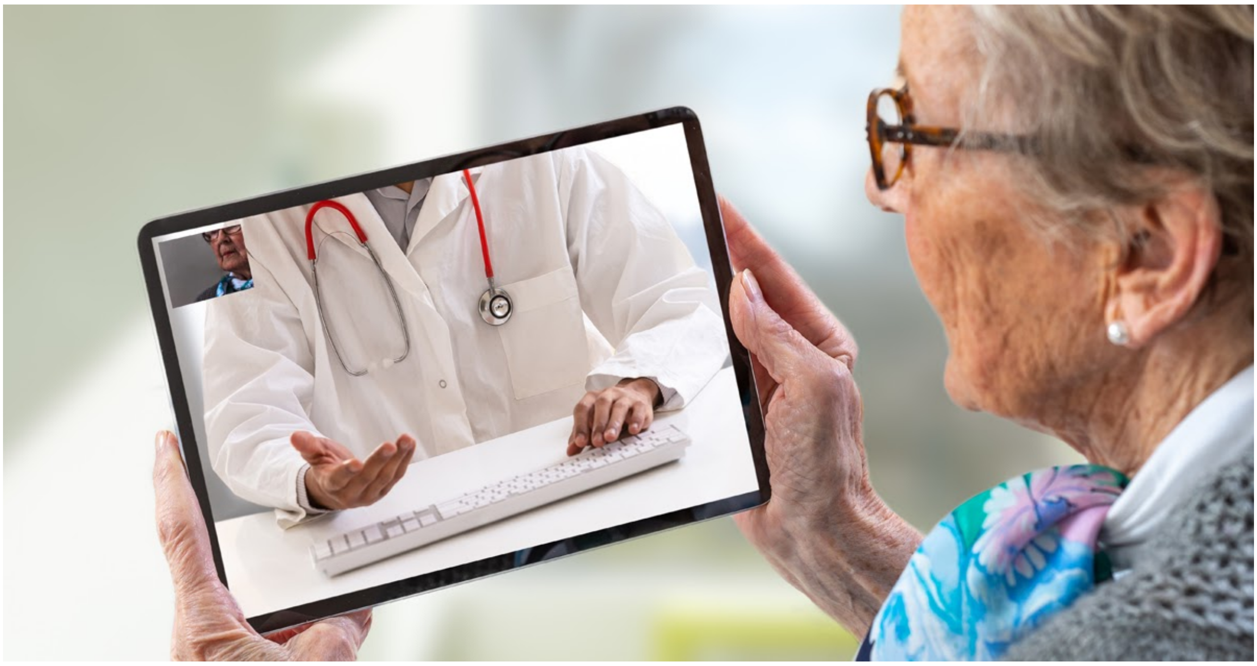

1. Please choose your language; Sila pilih bahasa anda；请选择您的语言 *

Mark only one oval.

- English *Skip to question 2*
- Bahasa Malaysia *Skip to question 8*
- 中文 *Skip to question 14*

RESEARCH TITLE
Effect, Acceptance, and Sustainability of a Hybrid Telemedicine Services among Geriatric Patients of Sarawak Heart Centre

SPONSOR
National Institute of Health, Ministry of Health, Malaysia

INTRODUCTION
You have been invited to take part in a research study to examine the effect, acceptance, and sustainability of a newly launched hybrid telemedicine services for geriatric patients named as "TeleG" at Sarawak Heart Centre.
This information sheet gives you a detailed description of this study and will help you to decide if you would like to participate. Please read this sheet thoroughly and ask any questions that may occur to you. Participation in this research is voluntary.
A total of 180 geriatric patients who have been seeking medical consultation at Sarawak Heart Centre will be recruited in this study.
This study has been approved by the Ethics Committee of MOH.

PURPOSE OF THE STUDY
This study aims to to examine the effect, acceptance, and sustainability of a newly launched hybrid telemedicine services for geriatric patients at Sarawak Heart Centre.

PARTICIPANTS CRITERIA
Elderly patients who have been seeking treatment at geriatric clinic of Sarawak Heart Centre will be screened for suitability before being invited to enrol in this study.

The inclusion criteria of are:
(1) patients who understand the benefits and limitations of TeleG and still agree to participate in the study;
(2) patients whose health conditions are deemed stable and suitable for TeleG by attending physicians:
i. Not a new case and must have been seen at least once physically, either at the ward or the geriatric clinic; or
ii. bed bound or impaired mobility; or
iii. clients of nursing home; or
iv. having logistic problem to visit the clinic physically (difficult to have transportation; reside at outskirt or rural areas; or reside in division or districts outside Kuching and Samarahan); or
v. require monitoring of side effects after initiation of drugs
(3) Patients who have a smart phone or a smart phone that support or perform video conferencing; for patients who rely on caregivers, their caregivers must have a smart phone that is able to support and perform video conferencing;
(4) Patients or caregivers whose residential area have stable internet connection;

The exclusion criteria are:
(1) Patients who visit the geriatric clinic for the first time;
(2) Patients who require continuous physical reviews;
(3) Patients who refuse to participate

This study will not include individuals who refuse to give consent to take part or unable to respond during the data collection period of time.

STUDY PROCEDURE
If you agree to take part in this study, the researcher will request you to provide a written informed consent. After that, you will be briefed about the registration of Zoom in order to perform online appointment and consultation. After completion of registration, you will be given an appointment date to meet your doctor through the Zoom.

Upon the first session of meeting your doctor through the Zoom, your doctor will give you medical consultation as usual during physical consultation. Upon the completion of consultations, the links to access the “patient acceptance form” and

Participant Information Sheet

"caregiver burden survey" electronically will be given to you and and your caregivers, through the Zoom chat function or Whatsapp or short message service. It takes approximately 10 minutes to answer all the questions. Your caregiver may help you to tick the answers of the "patient acceptance form" if such assistance is required.

After completing the survey, you will be given next clinic appointment (either another TeleG session or physical consultation as deemed appropriate by the attending geriatrician) and your participation in this study ends here and you will continue with treatment plans given by your geriatrician as usual.

RESPONSIBILITIES
It is important for you to answer all of the questions asked by the health care professionals who attend to you honestly and completely. The period of data collection will be ongoing for 12 months from the date you first join this study. There will be no additional cost incurred to you to participate in this study. A token of appreciation will be given to you upon the completion of your participation in this study.

RISKS
Participation in this study will not possess any potential risk, as this study does not involve any invasive procedure.

PARTICIPATION IN THE STUDY
Your taking part in this study is entirely voluntary. Respondents will not be required to sign in to an account in order to fill in the survey. If you decide to withdraw from the study midway, you could exit the site freely and no measures will be used to preserve the data you have filled in, thus all data will be destroyed.
Your participation also may be stopped by the research team without your consent if in any form you have violated the study eligibility criteria. The research team member will discuss with you if the matter arises.

The results of the study will be shared to the authorities and health care professionals. The results are general results without revealing any individual name or information. You will be informed if there is any new information that becomes available relevant to consent.

POSSIBLE BENEFITS
There may or may no be any benefits to you. The findings of this study would contribute to the extant body of knowledge on the impact, acceptance, and sustainability of a hybrid telemedicine services targeting elderly patients. Besides, it will provide insights to the policy makers, relevant authorities and healthcare professionals, on how to embark on the telemedicine that will sustain and have real impact on patients' health.

ENQUIRIES
If you have any question about this study or your rights, please contact;

The Secretary,
Medical Research & Ethics Committee,
Ministry of Health Malaysia,
Telephone no: 03-3362 8407/8205/8888.

Dr. Samuel Ting Chuo Yew (Ph.D.)
Principal investigator
Sarawak State Health Department
Phone no: 0168605496
Email: tingchuoyew@gmail.com

Dr Ling Jia Nee
Principal Investigator at Study Site
Physician/Geriatrician
Sarawak Heart Centre
Phone no: 0128564482
Email: lingjianee@gmail.com

CONFIDENTIALITY
Data collected will be recorded in offline subject data sheets after being retrieved from Google Form and all data in the Google Form will be destroyed. Your information will be kept confidential by the researchers and will not be made publicly available unless disclosure is required by law. Data obtained from this study does not identify you individually and will be published for knowledge purposes only.

Your original records or data may be reviewed by the individual involved, the Ethical Review Board for this study, and qualified monitors and auditors, governmental or regulatory authorities for the purpose of verifying the study procedures and/or data. Your information may be held and processed on a computer. Only research team members are authorized to access your information.

By providing implied consent, you authorize the record review, information storage, and data process described above.

CONFLICT OF INTEREST
All the investigators declare that they have no conflict of interest.

PUBLICATION POLICY
No personal information will be disclosed and subjects will not be identified when the findings of the survey are published. Permission from the Director-General of Health, Malaysia will be obtained prior to publication.

SIGNATURES
To be entered into the study, you or a legal representative must click agree to participate in this research.

Consent Form

Please read the consent form carefully before making any decision.

1. I have read all of the information in this Patient Information and Consent Form including any information regarding the risk in this study and I have had time to think about it.

2. All of my questions have been answered to my satisfaction.

3. I voluntarily agree to be part of this research study, to follow the study procedures.

4. I may freely choose to stop being a part of this study at any time.

5. I have received a copy of this Participant Information and Consent Form to keep for myself.

2. Do you agree to participate in this study? *

Mark only one oval.

- Yes
- No

3. Date of consent *

Example: January 7, 2019

4. Patient full name *

5. Patient IC *

6. Representative's name (If any)

7. Representative's IC (If any)
