# Supplementary material for: The use of a multi-disciplinary geriatric telemedicine service (TELEG) and its acceptance at a tertiary care centre in Malaysia
Source: BMC Geriatr. 2024 Feb 5;24:133. doi: 10.1186/s12877-024-04676-0 (PMC10845621; doi:10.1186/s12877-024-04676-0)
Supplement: Supplementary file 2 — Supplementary Material 2: Data collection form on health interventions prescribed through TELEG [file 12877_2024_4676_MOESM2_ESM.pdf]

Healthcare Team: Impact of TeleG

This survey form is to be filled by the attending geriatrician, pharmacist, and nurse respectively.

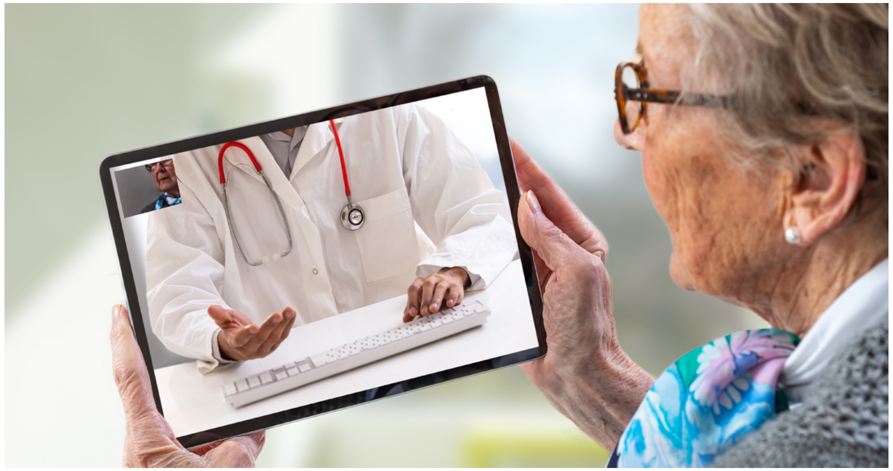

Socio-demographic (Filled by Nurse)

1. ID

This ID is the ID assigned during the recruitment of the patients

____

2. DATE

Date of filling this form (DDMMYYYY)

____

3. SEQUENCE

The sequence of current medical consultation

Mark only one oval.

- ◯ 1st
- ◯ 2nd
- ◯ 3rd
- ◯ 4th
- ◯ 5th
- ◯ 6th
- ◯ 7th
- ◯ 8th
- ◯ 9th
- ◯ 10th

INTERVENTIONS by attending geriatrician or medical officer

4. Description of interventions introduced by attending geriatrician or medical officer

____

____

____

____

____

Medication Adherence (Filled by Pharmacist)

The questions ask your current practice related to medicine taking in the past month

5. PHARMACIST

Pharmacist intervention notes

____
____
____
____
____

Nursing Care

6. NURSE

Please write down the nursing care notes

____
____
____
____
____

This content is neither created nor endorsed by Google.

Google Forms
