# Supplementary material for: The use of a multi-disciplinary geriatric telemedicine service (TELEG) and its acceptance at a tertiary care centre in Malaysia
Source: BMC Geriatr. 2024 Feb 5;24:133. doi: 10.1186/s12877-024-04676-0 (PMC10845621; doi:10.1186/s12877-024-04676-0)
Supplement: Supplementary file 3 — Supplementary Material 3: SUTAQ in English [file 12877_2024_4676_MOESM3_ESM.pdf]

For Patients: Acceptance and Impact of a Hybrid Telemedicine among Elderly Patients of Sarawak Heart Centre

* Required

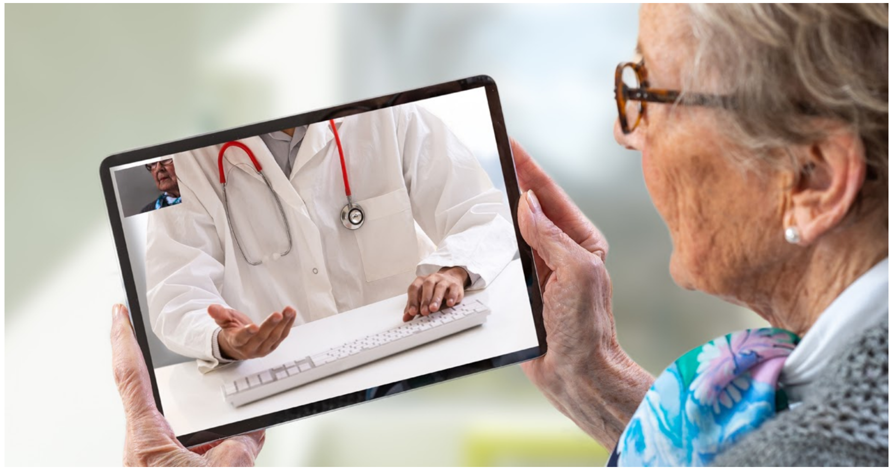

1. Please choose your language/ Sila pilih bahasa anda/ 请选择您的语言 *

Mark only one oval.

- English *Skip to question 2*
- Bahasa Malaysia *Skip to question 26*
- 中文 *Skip to question 50*

Acceptance towards TeleG

TeleG refers to the medical consultation conducted through Zoom or any other virtual platform, between your Dr and you.

2. A1 *

The TeleG has saved me time in that I did not have to visit my Dr or other health care professionals as often.

Mark only one oval.

- Strongly agree
- Moderately agree
- Mildly agree
- Mildly disagree
- Moderately disagree
- Strongly disagree

3. A2 *

The TeleG has interfered with my everyday routine.

Mark only one oval.

- Strongly agree
- Moderately agree
- Mildly agree
- Mildly disagree
- Moderately disagree
- Strongly disagree

4. A3 *

The TeleG has increased my access to health care professionals.

Mark only one oval.

- Strongly agree
- Moderately agree
- Mildly agree
- Mildly disagree
- Moderately disagree
- Strongly disagree

5. A4 *

The TeleG has helped me to improve my health.

Mark only one oval.

- Strongly agree
- Moderately agree
- Mildly agree
- Mildly disagree
- Moderately disagree
- Strongly disagree

6. A5 *

The TeleG has invaded my privacy.

Mark only one oval.

- Strongly agree
- Moderately agree
- Mildly agree
- Mildly disagree
- Moderately disagree
- Strongly disagree

7. A6 *

The TeleG has been explained to me sufficiently.

Mark only one oval.

- Strongly agree
- Moderately agree
- Mildly agree
- Mildly disagree
- Moderately disagree
- Strongly disagree

8. A7 *

The TeleG can be trusted to work appropriately.

Mark only one oval.

- Strongly agree
- Moderately agree
- Mildly agree
- Mildly disagree
- Moderately disagree
- Strongly disagree

9. A8 *

The TeleG has made me feel uncomfortable, e.g. physically or emotionally.

Mark only one oval.

- Strongly agree
- Moderately agree
- Mildly agree
- Mildly disagree
- Moderately disagree
- Strongly disagree

10. A9 *

I am concerned about the level of expertise of the individuals who monitor my status via the TeleG.

Mark only one oval.

- Strongly agree
- Moderately agree
- Mildly agree
- Mildly disagree
- Moderately disagree
- Strongly disagree

11. A10 *

The TeleG has allowed me to be less concerned about my health care.

Mark only one oval.

- Strongly agree
- Moderately agree
- Mildly agree
- Mildly disagree
- Moderately disagree
- Strongly disagree

12. A11 *

The TeleG has made me more actively involved in my health.

Mark only one oval.

- Strongly agree
- Moderately agree
- Mildly agree
- Mildly disagree
- Moderately disagree
- Strongly disagree

13. A12 *

The TeleG makes me worried about the confidentiality of the private information being exchanged through it.

Mark only one oval.

- Strongly agree
- Moderately agree
- Mildly agree
- Mildly disagree
- Moderately disagree
- Strongly disagree

14. A13 *

The TeleG allows the people looking after me, to better monitor me and my condition.

Mark only one oval.

- Strongly agree
- Moderately agree
- Mildly agree
- Mildly disagree
- Moderately disagree
- Strongly disagree

15. A14 *

I am satisfied with the TeleG

Mark only one oval.

- Strongly agree
- Moderately agree
- Mildly agree
- Mildly disagree
- Moderately disagree
- Strongly disagree

16. A15 *

The TeleG can be/should be recommended to people in a similar condition to mine.

Mark only one oval.

- Strongly agree
- Moderately agree
- Mildly agree
- Mildly disagree
- Moderately disagree
- Strongly disagree

17. A16 *

The TeleG can be a replacement for my regular health care.

Mark only one oval.

- Strongly agree
- Moderately agree
- Mildly agree
- Mildly disagree
- Moderately disagree
- Strongly disagree

18. A17 *

The TeleG can certainly be a good addition to my regular health care.

Mark only one oval.

- Strongly agree
- Moderately agree
- Mildly agree
- Mildly disagree
- Moderately disagree
- Strongly disagree

19. A18 *

The TeleG is not as suitable as regular face to face consultations with the people looking after me.

Mark only one oval.

- Strongly agree
- Moderately agree
- Mildly agree
- Mildly disagree
- Moderately disagree
- Strongly disagree

20. A19 *

The TeleG has made it easier to get in touch with health care professionals.

Mark only one oval.

- Strongly agree
- Moderately agree
- Mildly agree
- Mildly disagree
- Moderately disagree
- Strongly disagree

21. A20 *

The TeleG interferes with the continuity of the care I receive (i.e. I do not see the same care professional each time).

Mark only one oval.

- Strongly agree
- Moderately agree
- Mildly agree
- Mildly disagree
- Moderately disagree
- Strongly disagree

22. A21 *

I am concerned that the person who monitors my status, through the TeleG, does not know my personal health care history.

Mark only one oval.

- Strongly agree
- Moderately agree
- Mildly agree
- Mildly disagree
- Moderately disagree
- Strongly disagree

23. A22 *

The TeleG has allowed me to be less concerned about my health status.

Mark only one oval.

- Strongly agree
- Moderately agree
- Mildly agree
- Mildly disagree
- Moderately disagree
- Strongly disagree

Impact

24. In total, how much time you spent (minute) on this virtual medical consultation with your doctor? (From preparing until the end of the consultation. If you need to travel to another place to have this virtual medical consultation, you need to include the time you travel thru and fro) *

____

25. In the past, when you visit the Sarawak Heart Centre to have physical medical consultations with your doctor, how much time you spent (hour) from leaving your home until you are back at your home? *

____
